# An uncommon cause of progressive visual loss in a heavy smoker

**DOI:** 10.11604/pamj.2015.22.47.7864

**Published:** 2015-09-18

**Authors:** Dimitris Koufakis, Dimitrios Konstantopoulos, Theocharis Koufakis

**Affiliations:** 1 Larissa Macula Center, Larissa, Greece; 2 Department of Internal Medicine, General Hospital of Larissa, Larissa, Greece

**Keywords:** Lung cancer, metastasis, choroid

## Abstract

Loss of vision due to eye metastasis is generally uncommon, representing an ophthalmological diagnostic and therapeutic challenge. We here report a case of a smoker patient finally diagnosed with lung cancer, whose initial symptom was visual loss due to choroidal metastasis. Given that the majority of subjects presenting with uveal metastasis have already developed other distant metastases as well, a complete diagnostic work-up of these patients is always required. Despite being rare, eye metastasis from a lung malignancy should always be suspected in smokers presenting with progressive vision deterioration.

## Introduction

Gradual loss of vision is one of the commonest reasons for which patients present to the ophthalmologist. It may have various causes which can be divided into two main categories, according to the reversibility of the visual loss. Reversible conditions include, among others, cataract, refractive error and diabetic macular œdema, while irreversible ones are optic atrophy, glaucoma, retinitis pigmentosa and age-related macular degeneration [1]. Loss of vision due to eye metastasis is generally uncommon, representing an ophthalmological diagnostic and therapeutic challenge.

## Patient and observation

**Figure 1:**
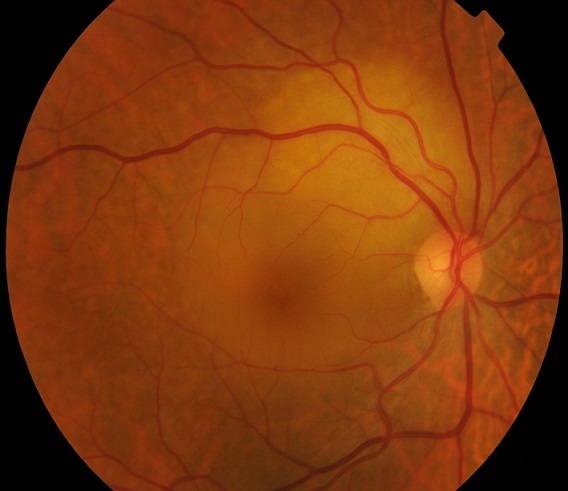
Color funds photograph of the Right Eye (RE) showing a yellowish juxtpapillary subretinal mass with associated serous macular detachment

**Figure 2:**
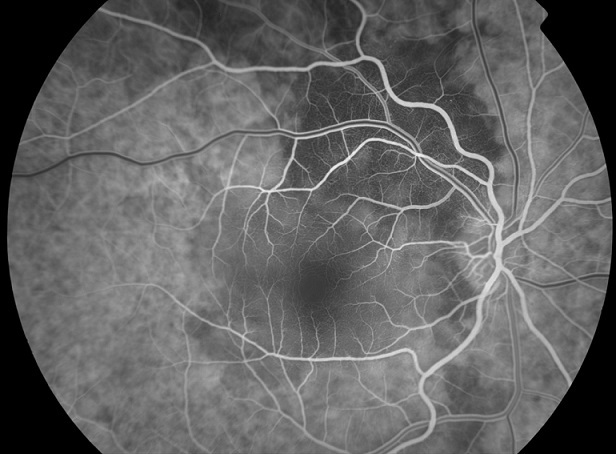
Fundus Fluorescein Angiography (FFA) of the RE, early frames, showing hypofluorescence of the mass as a result of transmission defect from the choroidal circulation

**Figure 3:**
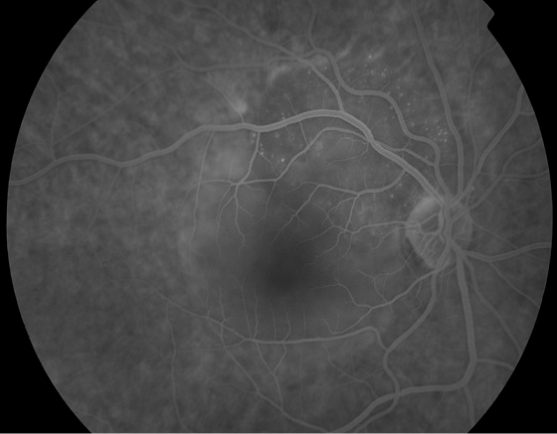
FFA of the RE, late frames, showing hyperfluorescence of the mass due to dye leakage

**Figure 4:**
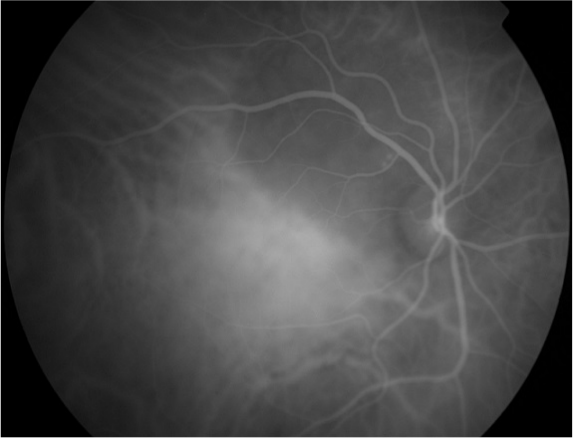
Indocyanine Green Angiography (ICG) of the RE, late frames. The lesion is hypofluorescent throughout the study

A 60-year-old female patient, with history of smoking habit (80 packyears), presented with complaints of progressive visual loss from her right eye. Her symptoms had started approximately two months prior to presentation. Best-corrected visual acuity was 6/24 in the right eye and 6/5 in the left eye. Anterior segment examination was unremarkable in both eyes. Intraocular pressure was within normal limits, there was no relative afferent pupillary defect and the vitreous was clear in both eyes. Dilated fundus examination showed an elevated juxtpapillary yellowish mass in the right eye with an associated macular detachment (Figure 1), which was confirmed by Optical Coherence Tomography (OCT). The differential diagnosis mainly included amelanotic choroidal melanoma, circumscribed choroidal hemangioma and choroidal metastasis. Fundus Fluorescein Angiography (FFA) (Figure 2, Figure 3), Indocyanine Green Angiography (IGA) (Figure 4) and B scan were performed and confirmed the presence of choroidal metastasis. Subsequently, the patient was referred to the Internal Medicine department, where she underwent Computed Tomography (CT) scan of the brain, chest and abdomen, bone scan, as well as gastroscopy and colonoscopy. The imaging methods revealed a lesion located at the upper lobe of the right lung and pathologically enlarged local lymph nodes. Moreover, numerous bone metastases were detected. The patient was subjected to bronchoscopy and histological results from the biopsy taken from the tumor were compatible with small cell carcinoma. She died three months later despite having received appropriate chemotherapy treatment.

## Discussion

Small cell lung cancer (SCLC) is a highly metastatic type of lung cancer, which is etiologically directly associated with tobacco use [2]. It has been estimated that almost 70% of the patients suffering from SCLC are presenting with locally advanced or metastatic disease at the time of diagnosis [3], with most frequent sites being liver, bones, brain, lungs and adrenal glands [4]. Choroidal metastases, the most common malignant intraocular lesions, occur mainly in lung and breasts carcinomas [5]. However, rarely, they have been reported in other types of cancer. Given that the majority of subjects presenting with uveal metastasis have already developed other distant metastases as well [6], a complete diagnostic work-up of these patients is always required. Unfortunately, overall survival time in these cases does not seem to exceed 12 months [6]. Methods for the diagnosis and investigation of ocular metastases include ultrasonography, fluorescein angiography, computed tomography, magnetic resonance imaging (MRI), fine-needle aspiration and wedge biopsy. With regard to therapeutic options, external beam radiotherapy remains the treatment of choice [5]. Bevacizumab, a monoclonal antibody, has been used in the treatment of choroidal metastasis and relevant studies have demonstrated promising results [7].

## Conclusion

In our patient, visual loss was the initial presentation of lung cancer and the symptom that finally led to the diagnosis. In conclusion, despite being rare, eye metastasis from a lung malignancy should always be suspected in smokers presenting with progressive vision deterioration.
